# Significant association between functional microRNA polymorphisms and head and neck cancer susceptibility: a comprehensive meta-analysis

**DOI:** 10.1038/srep12972

**Published:** 2015-08-17

**Authors:** Yu-Ming Niu, Xin-Ya Du, Ming-Yi Lu, Qiong-Li Xu, Jie Luo, Ming Shen

**Affiliations:** 1 Department of Stomatology and Center for Evidence-Based Medicine and Clinical Research, Taihe Hospital, Hubei University of Medicine, 32 South Renmin Road, Shiyan 442000, China; 2 Department of Stomatology, People’s Hospital of New District Longhua Shenzhen, 2 East Jianshe Road, Shenzhen 518109, China; 3 Department of Oral and Maxillofacial Surgery, Chung Shan Medical University Hospital, No. 110, Sec. 1, Chien-Kuo N. Rd, Taichung 40201, China; 4 Department of Neurosurgery and Evidence-Based Medicine Center, Taihe Hospital, Hubei University of Medicine, 32 South Renmin Road, Shiyan 442000, China; 5 Jiangsu Key Laboratory of Oral Diseases, Nanjing Medical University; Department of Dental Implant, Affiliated Hospital of Stomatology, Nanjing Medical University, No. 140 Hanzhong Road, Nanjing 210029, China

## Abstract

Molecular epidemiological studies have showed a closer association between microRNA polymorphisms with and head and neck cancer (HNC) risk. But the results of these studies were inconsistent. We performed this meta-analysis to clarify the associations between microRNA polymorphisms and HNC risk. Four electronic databases (PubMed, Embase, CNKI, and Wanfang) were searched. Odds ratios (ORs) with 95% confidence interval (CIs) were calculated to assess the association between microRNA-146a rs2910164 G > C, microRNA-196a2 rs11614913 C > T, microRNA-149 rs2292832 C > T, microRNA-499 rs3746444 A > G polymorphisms and HNC risk. Heterogeneity, publication bias and sensitivity analysis were conducted to guarantee the statistical power. Overall, 11 selected articles involving 16100 subjects were included in this meta-analysis. Significantly increased risk between microRNA-146a rs2910164 G > C polymorphism and HNC risk were observed in Caucasian population (GC vs. GG: OR = 1.31, 95%CI = 1.01–1.68; GC + CC vs. GG: OR = 1.26, 95%CI = 1.02–1.57). For microRNA-196a2 rs11614913 C > T, similarly increased risk were also found in Asian population (T vs. C, OR = 1.14, 95%CI = 1.04–1.25; TT vs. CC, OR = 1.33, 95%CI = 1.09–1.61; CT + TT vs. CC OR = 1.32, 95%CI = 0.99–1.76; TT vs. CC + CT, OR = 1.14, 95%CI = 0.99–1.33). In addition, no significant association was detected between microRNA-149 rs2292832 C > T and microRNA-499 rs3746444 A > G polymorphism and HNC risk. This meta-analysis demonstrates that microRNA polymorphisms are associated with HNC development based on ethnicity diversity.

Head and neck cancer (HNC) is the sixth most common malignancy worldwide and comprises a variety of epithelial malignancies involving the oral cavity, nasal cavity, thyroid, pharynx, and larynx^1^. Approximately 633,000 new cases and 355,000 deaths were reported in 2008, resulting in severe disability, reduced the quality of life, and a poor survival rate, as well as an increased economic burden on individuals and society^2^,^3^. Various factors, such as lifestyle habits (tobacco and alcohol consumption), viral infection (human papillomavirus (HPV)) and oral hygiene have been proven to contribute to the development of HNC^4^,^5^,^6^,^7^. However, the factors contributing to susceptibility are still being explored. Progress has been made recently, but the treatment and prognosis for HNC are not yet satisfactory.

To date, many molecular epidemiological studies have shown that genetic factors may play an important role in tumorigenesis, and the genetic predisposition is gaining increasing attention^1^,^8^,^9^. MicroRNAs are short, single-stranded, noncoding RNAs that are 20–22 nucleotides long and they participate in the post-transcriptional regulation of gene expression. They are critical regulators of various fundamental biological processes such as proliferation, differentiation, apoptosis^10^,^11^,^12^. Researchers have found that microRNAs play an important role in the development of human cancers such as breast, lung, cervical, gastric, and colorectal cancer^13^,^14^,^15^,^16^. MicroRNAs could function as oncogenes or tumor suppressors and regulate the cell proliferation and apoptosis processes, resulting in solid cancer formation owing to the abnormal accumulation of tumor cells.

Recently, an increasing number of studies have focused on the association between genetic mutations and disease susceptibility^17^. Single-nucleotide polymorphisms (SNPs) are thought to be one of the most important genetic variations in the human genome^18^. SNPs are variations in the DNA that are spaced throughout human chromosomes, and they are genetic variations that arise from single nucleotide mutations. The DNA sequence variation occurs commonly within a population in which a single nucleotide (A, T, C or G) in the genome differs between members of a biological species or paired chromosomes^19^. Polymorphisms can be found in different aspects of the microRNA signal pathway such as pre-microRNA, mature microRNA, and the target or binding gene sites^20^,^21^. All mutations would interfere with the translation of messenger RNA (mRNA) at the post-transcriptional level and regulate the protein expression of target genes, possibly leading to abnormal biological metabolism and increase risk of cancer development^21^,^22^. The four most common polymorphisms are microRNA-146a rs2910164, which is located on chromosome 5q34 with a nucleotide mutation from G to C^23^, microRNA-196a2 rs11614913, which is located on chromosome 12q13.13 with a nucleotide mutation from C to T^24^, microRNA-149 rs2292832, which is located on chromosome 2q37.3 with a nucleotide mutation from C to T^25^, and microRNA-499 rs3746444, which is located on chromosome 20q11.22 with a nucleotide mutation from A to G^26^.

In 2008, Jazdzewski *et al.*^27^ conducted found that GC heterozygous of microRNA-146a rs2910164 G > C may be an increased risk factor for acquiring papillary thyroid carcinoma (PTC). Today, all four SNPs have been reported to be associated with HNC risk, but the results have been conflicting. Therefore, a comprehensive meta-analysis involving the related publications was performed to assess the possible association between microRNA polymorphisms and HNC susceptibility.

## Methods

### Search strategy

Four electronic databases (Pubmed, Embase, CNKI, and Wanfang) were searched using the following terms: “microRNA”, “miRNA”, “head and neck cancer”, “polymorphism”, and “variant”, up to December 1, 2014. The combined phrases for all genetic studies on the association between HNC and microRNA polymorphisms were also used. Only studies written in English and Chinese were selected.

### Study selection

All selected studies fulfilled the following inclusion criteria: (1) case-control design focus on HNC; (2) research on microRNA polymorphisms; and (3) adequate genotype data (or data available to calculate) to assess the odds ratio (OR) and 95% confidence interval (CI). The exclusion criteria included: (1) review articles; (2) case reports; (3) results without the research polymorphisms or outcome data; (4) animal model research; and (5) repeated or overlapping publications with the same author or team were deleted according to the publication date or sample size.

### Data extraction

Two reviewers (Niu and Du) independently collected the data for analysis, including the first author’s name, publication year, sources of controls, study country/region, ethnicity of participants (such as Asian or Caucasian), genotyping method, and number of genotypes in HNC cases and controls. A third reviewer was introduced (Lu) to adjust all discrepancies during the analysis for consistency. The Hardy-Weinberg equilibrium (HWE) was calculated based on the genotypes of the controls.

### Statistical analysis

ORs with 95% CIs were calculated to evaluate the strength of the association between the four polymorphisms and HNC risk. For the microRNA-146a rs2910164 G > C polymorphism, the pooled ORs were obtained for the allele contrast (C vs. G), co-dominant model (GC vs. GG, CC vs. GG), dominant model (GC + CC vs. GG), and recessive model (CC vs. GG + GC)^28^,^29^. Similar genetic models were also assessed for the microRNA-149 rs2292832 C > T, microRNA-196a2 rs11614913 C > T and microRNA-499 rs3746444 A > G variants. Subgroup analyses of ethnicity, study design, cancer location (type) and genotyping methods were also submitted to statistical testing. Heterogeneity was assessed with the Cochran’s Q statistic and *I*^2^ method^30^, ORs estimation was calculated with a fixed-effects model (the Mantel-Haenszel method) when the P value was more than 0.10 or *I*^2^ was less than 40%; otherwise, a random-effects model (DerSimonian and Laird method) was adopted. A furthermore meta-regression was conducted to analyze the existed heterogeneity^31^. Cumulative meta-analyses and sensitivity analyses were conducted to evaluate the stability of the results by removal of each study sequentially for each polymorphism. The potential publication bias of the literature was analyzed by Egger’s linear regression and Begg’s funnel plots^32^. Statistical analysis was performed using STATA version 11.0 (Stata Corporation, College Station, TX, USA) with two-sided P values and *P* < 0.05 considered statistically significant.

## Results

### Study characteristics

**Figure 1:**
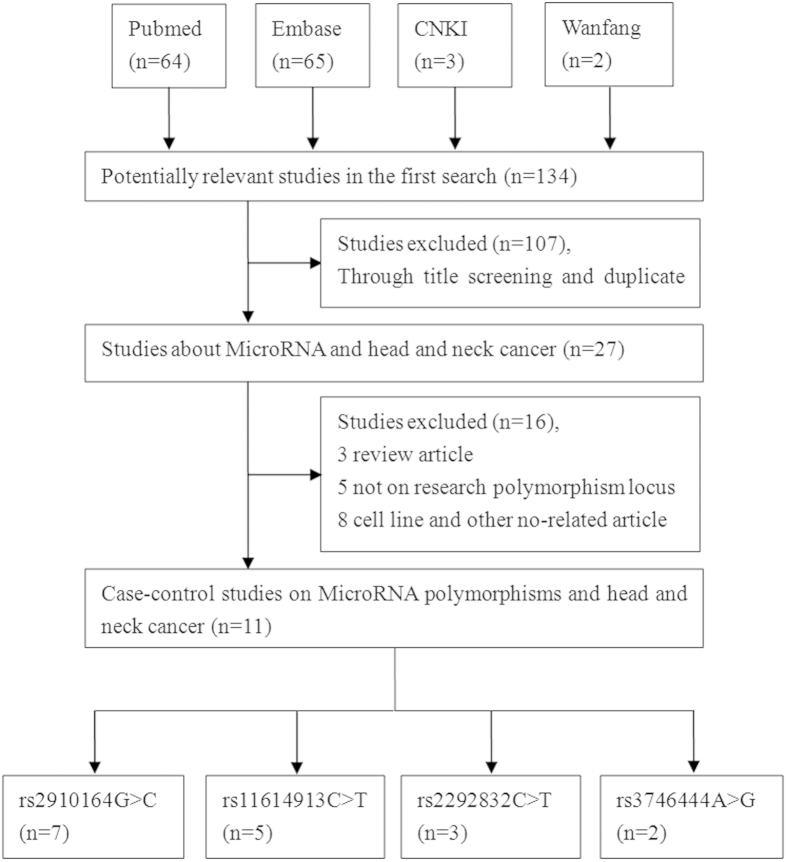
Flow diagram of the study selection process.

A total of 134 relevant studies were identified from a systematic literature search. The search procedure is shown in Fig. 1. Following the study selection criteria, 107 studies were excluded in the first step of title and duplicate screening step, and 16 studies were subsequently excluded from our research due to various deficiencies(3 were reviews, 5 were not on the research polymorphism locus, and 8 were focused on cell line and others). In total, 11 eligible articles were selected with adequate data^27^,^33^,^34^,^35^,^36^,^37^,^38^,^39^,^40^,^41^,^42^, including seven studies on microRNA-146a rs2910164 G > C^27^,^34^,^35^,^36^,^37^,^38^,^40^, five studies on microRNA-196a2 rs11614913 C > T^33^,^34^,^35^,^41^,^42^, three publications on microRNA-149 rs2292832 C > T^34^,^35^,^39^, and two studies on microRNA-499 rs3746444 A > G^34^,^35^, respectively. Four studies involved Caucasian populations^27^,^33^,^34^,^37^, and seven studies involved Asian populations^35^,^36^,^38^,^39^,^40^,^41^,^42^. Regarding the genotyping method, eight studies used the Applied Biosystems^27^,^33^,^35^,^36^,^39^,^40^,^41^,^42^, two studies adopted polymerase chain reaction-restriction fragment length polymorphism (PCR-RFLP)^34^,^35^, one study used PCR with two-pair primers method^37^ and another was conducted with the MassARRAY iPLEX platform^38^. Only one study deviated from the HWE analysis in microRNA-149 rs2292832 C > T polymorphism^35^. The detailed characteristics of the selected studies were summarized in Table 1.

### Quantitative analysis

#### For microRNA-146a rs2910164 G > C polymorphism

**Figure 2:**
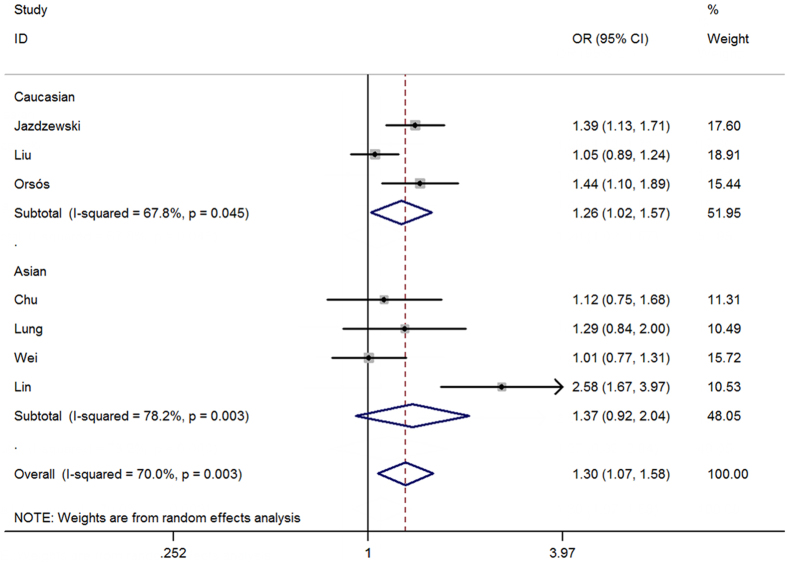
OR and 95% CIs for the associated between microRNA-146a rs2910164 G > C polymorphism with HNC risk in GC + CC vs. GG model.

**Figure 3:**
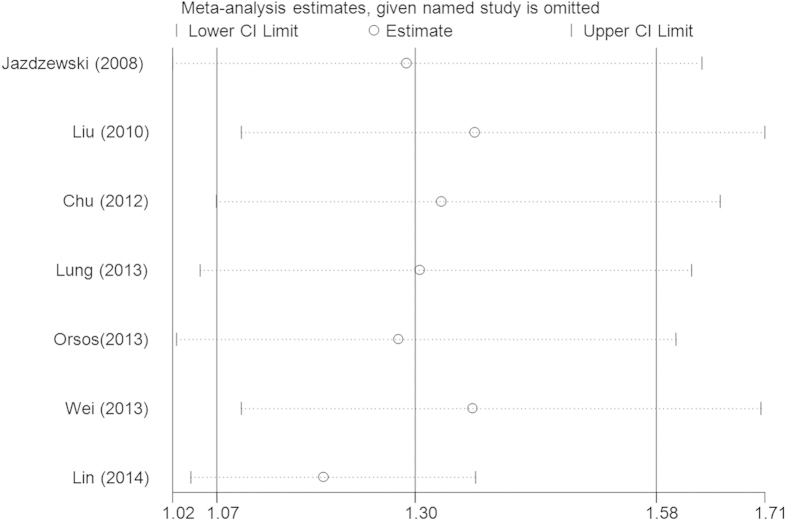
Sensitivity analysis through deleting each study to reflect the influence of the individual dataset to the pooled ORs in GC + CC vs. GG model of microRNA-146a rs2910164 G > C polymorphism.

**Figure 4:**
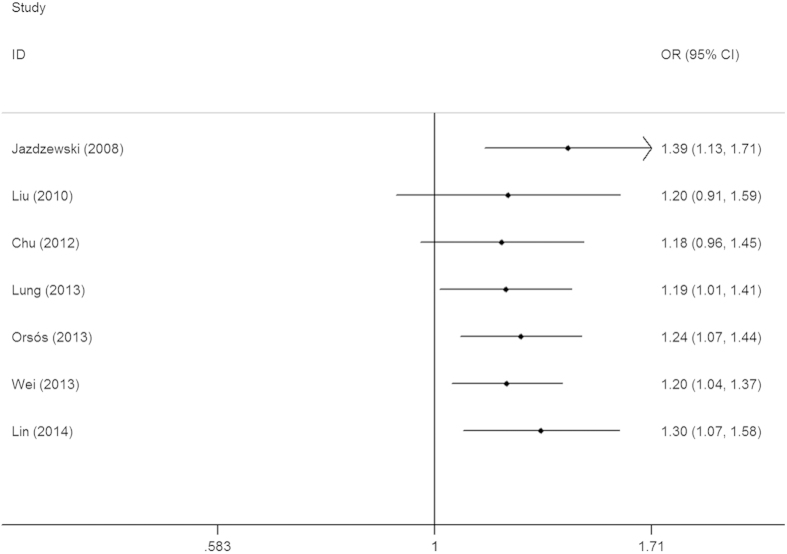
Cumulative meta-analyses according to publication year in GC + CC vs. GG model of microRNA-146a rs2910164 G > C polymorphism.

**Figure 5:**
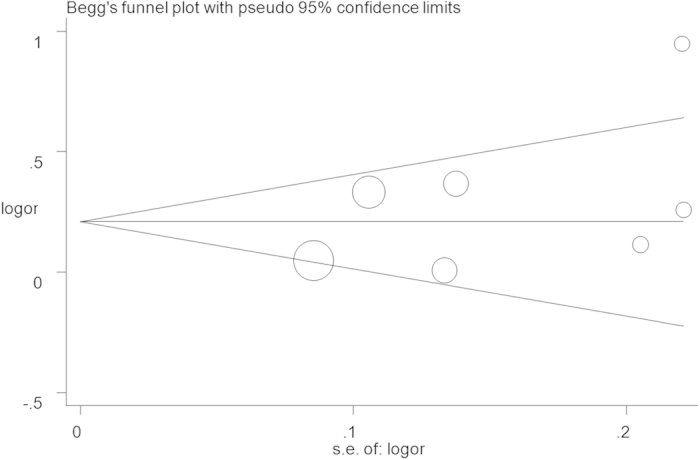
Funnel plot analysis to detect publication bias for GC + CC vs. GG model of microRNA-146a rs2910164 G > C. Circles represent the weight of studies.

Seven eligible studies with 3,841 cases and 7,900 controls focused on microRNA-146a rs2910164 G > C. The results of the combined analyses revealed a significantly increased risk for HNC risk in the genotype mutation genetic models(C vs. G: OR = 1.20, 95%CI = 1.04–1.39, *P* = 0.01, *I*^2^ = 77.9%; GC vs. GG: OR = 1.27, 95%CI = 1.05–1.55, *P* = 0.02, *I*^2^ = 68.4%; GC + CC vs. GG: OR = 1.30, 95%CI = 1.07–1.58, *P* = 0.01, *I*^2^ = 70.0%, Fig. 2) (Table 2). Heterogeneities existed in all five models. Meta-regression analyses and stratified analyses were conducted, but no critical factors were found to explain these heterogeneities. In the subgroup analyses by ethnicity and control design, significantly increased risks were also found in the Caucasian population (GC vs. GG: OR = 1.31, 95%CI = 1.01–1.68, *P* = 0.04, *I*^2^ = 75.3%; GC + CC vs. GG: OR = 1.26, 95%CI = 1.02–1.57, *P* = 0.03, *I*^2^ = 68.7%) and with some others gene models (Table 2). Furthermore, some significantly increased risks were also observed in the subgroup analysis with genotyping method of Applied Biosystems (Table 2). Sensitivity analysis showed that no single study qualitatively changed the pooled ORs, indicating that the results of this meta-analysis were highly stable (Fig. 3 fordominant model). A cumulative analysis by publication date showed that the results gradually showed a positive association beginning with a study by Lung *et al.*^36^ published in 2013 (Fig. 4 for dominant model). Funnel plot and Egger’s test were performed to estimate the publication bias of the literature, which did not reveal any asymmetrical evidence (Fig. 5 fordominant model). The results were further supported by the analysis of the data with Egger’s test (C vs. G: *P* = 0.05; GC vs. GG: *P* = 0.57; CC vs. GG: *P* = 0.57; GC + CC vs. GG: P = 0.24; CC vs. GG + GC: *P* = 0.87).

#### For microRNA-196a2 rs11614913 C > T

Five publications with 3,534 cases and 3,564 controls reported the association between microRNA-196a2 rs11614913 C > T polymorphisms and HNC risk. Overall, significant results were observed in the allele contrast model (T vs. C, OR = 1.10, 95%CI = 1.03–1.19, *P* = 0.01, *I*^2^ = 0%) and co-dominant model (TC vs. CC, OR = 1.21, 95%CI = 1.04–1.41, *P* = 0.01, *I*^2^=2.5%) (Table 2). Subsequent stratified analysis according to ethnicity and increased risks were found in an Asian population (T vs. C, OR = 1.14, 95%CI = 1.04–1.25, *P* = 0.01, *I*^2^ = 0%; TT vs. CC, OR = 1.33, 95%CI = 1.09–1.61, *P* < 0.01, *I*^2^ = 0%; CT + TT vs. CC OR = 1.32, 95%CI = 0.99–1.76, *P* = 0.06, *I*^2^ = 69.7%; TT vs. CC + CT, OR = 1.14, 95%CI = 0.99–1.33, *P* = 0.08, *I*^2^ = 44.8%) (Table 2). Moreover, the similarly increased cancer risks were found in the allele contrast, co-dominant (TT vs. CC) and recessive (CC vs. GG + GC) models with genotyping method of Applied Biosystems. Publication bias analysis was also conducted, and the funnel plots were symmetric with Egger’s test approved (T vs. C: *P* = 0.26; TC vs. CC: *P* = 0.19; TT vs. CC: *P* = 0.25; TC + TT vs. CC: *P* = 0.36; TT vs. CC + TC: *P* = 0.82).

#### For microRNA-149 rs2292832 C > T and microRNA-499 rs3746444 A > G

Three studies involving 1,852 cases and 1,677 controls and two studies with 1,579 cases and 1,555 controls were included in the microRNA-149 rs2292832 C > T polymorphism, microRNA-499 rs3746444 A > G and HNC risk research, respectively. No significant associations were found in all models for the two SNPs loci (Table 2). The subgroup analyses based on ethnicity, control design and genotyping methods were conducted and no significant associations were found.

## Discussion

HNC is one of the most common malignant diseases in the world. Many treatment measures have been conducted in recent decades. However, morbidity and mortality are still high, and the prognosis is still poor. To date, with elucidation of the pathogenesis mechanism for interactions between microRNAs and cancer development, an increasing amount of attention has been paid to the association between the SNPs of microRNAs and HNC risks.

In 2008, Jazdzewski *et al.*^27^ reported the first significant increased association between the GC heterozygous and PTC risk recessive model (OR = 1.62, 95%CI = 1.3–2.0). Since then, a series of molecular epidemiological studies have been conducted, but the conclusions were inconsistent. In this meta-analysis, we investigated the associations between microRNA-146a rs2910164 G > C, microRNA-196a2 rs11614913 C > T, microRNA-149 rs2292832 C > T, and microRNA-499 rs3746444 A > G polymorphisms and HNC susceptibility on the basis of eleven selected case-control studies. Both microRNA-146a rs2910164 G > C and microRNA-196a2 rs11614913 C > T polymorphisms showed a significant association with HNC risk based on a large sample size and greater number of studies. In the subgroup analysis based on ethnic diversity, we observed an increased risk for the microRNA-146a rs2910164 G > C polymorphism and HNC in the Caucasian population. Moreover, similar results also indicated that the microRNA-196a2 rs11614913 C > T may play a risk role in the development of HNC in the Asian population. In the past few decades, some studies have shown that different distribution of genotype existed in different ethnicity and influenced the disease susceptibility. Our meta-analysis also indicated that the ethnicity differences may be the most critical factor resulting in HNC susceptibility among the Asian and Caucasian populations. Furthermore, it is worth noting that some significantly increased risks were observed in these analyzed results with the genotyping method of Applied Biosystems, but not PCR-RFLP. This consistency of results indicated that the genotyping method of Applied Biosystems was more useful to improve the accuracy of an experiment and to reduce some possible errors.

To our knowledge, this is the first quantitative assessment focused on the association between microRNA polymorphisms and HNC risk specially. Eleven articles involving 6,069 cases of HNC cases and 10,031controls were included. Even though number of studies included in this meta-analysis was small, we believe that the findings can help to explain the association between microRNA polymorphisms and HNC risk. First, the genotype distributions in the controls of four selected SNP loci were all mostly consistent with HWE. Second, all five analysis comparison patterns were conducted, and the significant association were always consistent. Third, Egger’s test and Begg’s funnel plots proved that there was no apparent publication bias was existence in our meta-analysis. All these data would guarantee the strength of our results.

However, there were some limitations in this meta-analysis. First, heterogeneity existed and was especially high for the microRNA-146a rs2910164 G > C polymorphism. To our knowledge, much variability among studies in a systematic review is termed heterogeneity. There were three most important sources of variability between the studies; i.e., clinical diversity (sometimes called clinical heterogeneity), methodological diversity (sometimes called methodological heterogeneity) and statistical heterogeneity^43^. We followed convention and referred to statistical heterogeneity simply as heterogeneity. In regards to the statistical heterogeneity in our analysis, factors such as the diversity of cancer type, classification of disease severity, environment and personal habits could influence the results. Furthermore, the diversity of genotyping methods among the included studies could bring about the existence of heterogeneity, which also would partly change the analyzed results. Second, the number of studies describing each polymorphism was limited, influencing the statistical power of our meta-analysis. Third, environmental factors such as smoking, drinking, and HPV infection have been shown to influence the development of HNC, and the status of local tumor invasion and lymph node metastasis may be influenced by the genetic mutation. However, in our meta-analysis, the interaction between the genetic mutation, environment factors, disease stage, and HNC susceptibility could not be conducted due to the data deficiency.

In conclusion, this meta-analysis indicated that two functional polymorphisms of microRNA-146a rs2910164 G > C and microRNA-196a2 rs11614913 C > T may play an important role in the development of HNC, especially considering ethnicity diversity. Further investigation into the relationship between microRNA polymorphisms, environmental factors, and HNC susceptibility is still needed.

## Additional Information

**How to cite this article**: Niu, Y.-M. *et al.* Significant association between functional microRNA polymorphisms and head and neck cancer susceptibility: a comprehensive meta-analysis. *Sci. Rep.* **5**, 12972; doi: 10.1038/srep12972 (2015).

## Figures and Tables

**Table 1:** Characteristics of case-control studies on microRNA polymorphisms and HNC risk included in the meta-analysis.

First author	Year	Country/Region	Racial	Source of controls	Case	Control	Genotype distribution							Genotyping methods	*P*for HWE^a^	Location
							Case				Control					
microRNA-146a rs2910164 G > C																
							GG	GC		CC	GG	GC	CC			
Jazdzewski	2008	Europe/USA	Caucasian	Healthy	608	901	305	287		16	526	320	55	Applied Biosystems	0.50	Thyroid
Liu	2010	USA	Caucasian	Population	1109	1130	630	411		68	655	405	70	PCR-RFLP	0.49	HN
Chu	2012	China	Asian	Hospital	470	425	54	242		174	54	196	175	PCR-RFLP	0.94	HN
Lung	2013	China	Asian	Healthy	229	3776	24	88		117	497	1807	1472	Applied Biosystems	0.12	NP
Orsós	2013	Hungary	Caucasian	Hospital	468	468	284	168		16	323	136	9	PCR with two-pair primers	0.22	HN
Wei	2013	China	Asian	Population	753	760	136	323		294	138	345	277	MassARRAY iPLEX platform	0.09	Thyroid
Lin	2014	China	Asian	Population	204	440	31	110		63	139	220	81	Applied Biosystems	0.71	Laryngeal
microRNA-196a2 rs11614913 C > T																
							CC	CT		TT	CC	CT	TT			
Christensen	2010	USA	Caucasian	Population	484	555	182	302^a^			188	367^a^		Applied Biosystems	NA	HN
Liu	2010	USA	Caucasian	Population	1109	1130	350	565		194	383	545	202	PCR-RFLP	0.74	HN
Chu	2012	China	Asian	Hospital	470	425	57	277		136	87	206	132	PCR-RFLP	0.69	HN
Roy	2014	India	Asian	Hospital	451	448	218	187		46	242	168	38	Applied Biosystems	0.25	Oral
Li	2014	China	Asian	Population	1020	1006	209	489		322	218	518	270	Applied Biosystems	0.30	NP
microRNA-149 rs2292832 C > T																
							CC	CT		TT	C/C	CT	TT			
Liu	2010	USA	Caucasian	Population	1109	1130	580	441		88	586	445	99	PCR-RFLP	0.27	HN
Chu	2012	China	Asian	Hospital	470	425	37	88		345	26	84	315	Applied Biosystems	< 0.01	HN
Tu	2012	China	Asian	Hospital	273	122	20	129		124	21	52	49	Applied Biosystems	0.27	HN
microRNA-499 rs3746444 A > G																
							AA		GG	AA	AG	GG				
Liu	2010	USA	Caucasian	Population	1109	1130	745		309	55	710	366	54	PCR-RFLP	0.44	HN
Chu	2012	China	Asian	Hospital	470	425	339		119	12	356	66	3	PCR-RFLP	0.98	HN

MAF: Minor allele frequency in control group.

NP: Nasopharyngeal; HN: head and neck.

Population: Population controls Hospital: Hospital controls Healthy: Healthy controls.

^a^HWE in control.

**Table 2:** Summary ORs and 95% CI of microRNA polymorphisms and HNC risk.

Locus	N*	No. of case/control	OR	95% CI	*P*	*I*^2^(%)^a^	OR	95% CI	*P*	*I*^2^(%)^a^	OR	95% CI	*P*	*I*^2^(%)^a^	OR	95% CI	*P*	*I*^2^(%)^a^	OR	95% CI	*P*	*I*^2^(%)^a^	
rs2910164 G > C			C vs. G				GC vs. GG				CC vs. GG				GC + CC vs.GG				CC vs. GG + GC				
Total	7	3841/7900	**1.20**	**1.04**–**1.39**	**0.01**	**77.9**	**1.27**	**1.05**–**1.55**	**0.02**	**68.4**	1.27	0.86–1.88	0.22	80.8	**1.30**	**1.07**–**1.58**	**0.01**	**70.0**	1.12	0.83 –1.53	0.46	82.4	
Ethnicity																							
Caucasian	3	2185/2499	1.15	0.98–1.33	0.08	56.0	**1.31**	**1.01**–**1.68**	**0.04**	**75.3**	0.96	0.50–1.83	0.89	75.0	**1.26**	**1.02**–**1.57**	**0.03**	**68.7**	0.87	0.42–1.79	0.71	80.2	
Asian	4	1656/5401	1.24	0.96–1.61	0.10	86.3	1.25	0.86–1.82	0.23	71.5	1.54	0.93–2.55	0.10	83.6	1.37	0.92–2.04	0.13	78.2	1.30	0.92–1.83	0.14	84.1	
Design																							
Population	3	2066/2330	1.23	0.93–1.62	0.15	88.2	1.24	0.85–1.83	0.27	81.3	1.15	0.79–2.88	0.21	88.9	1.33	0.86–2.06	0.19	87.1	1.27	0.89–1.83	0.19	75.8	
Hospital	2	938/893	1.13	0.78–1.65	0.52	84.0	**1.35**	**1.07**–**1.70**	**0.01**	**0.0**	1.29	0.76–2.53	0.45	54.6	**1.33**	**1.06**–**1.68**	**0.02**	**4.1**	1.11	0.54–2.28	0.78	66.4	
Healthy	2	837/4677	**1.25**	**1.02**–**1.53**	**0.03**	**57.3**	1.24	0.85–1.83	0.19	63.2	0.92	0.29–2.95	0.89	90.2	**1.37**	**1.14**–**1.66**	**0.01**	**0**	0.84	0.22–3.24	0.81	94.6	
Location																							
HN	3	2047/2023	1.09	0.89–1.32	0.41	69.8	**1.16**	**1.00**–**1.33**	**0.04**	**34.3**	1.08	0.83–1.39	0.57	18.8	1.18	0.95–1.46	0.13	48.7	0.95	0.76–1.15	0.52	36.5	
Thyroid	2	1361/1661	1.09	0.98–1.22	0.12	0	1.22	0.76–1.97	0.41	86.4	0.77	0.36–1.62	0.48	81.6	1.20	0.87–1.64	0.27	72.6	0.71	0.27–1.86	0.48	93.0	
Genotyping																							
AB	3	1041/5117	**1.40**	**1.08**–**1.81**	**0.01**	**79.2**	**1.53**	**1.06**–**2.20**	**0.02**	**66.0**	1.43	0.51–4.03	0.50	91.9	**1.63**	**1.12**–**2.39**	**0.01**	**71.2**	1.15	0.54–2.42	0.72	91.0	
PCR–RFLP	2	1579/1555	1.00	0.89–1.12	0.98	0	1.08	0.92–1.27	0.35	0	1.00	0.76–1.32	0.98	0	1.06	0.91–1.24	0.47	0	0.89	0.72–1.10	0.30	0	
rs11614913 C > T			T vs. C				CT vs. CC				TT vs. CC				CT + TT vs. CC				TT vs. CC + CT				
Total	5	3534/3564	**1.10**	**1.03**–**1.19**	**0.01**	0	1.25	0.98–1.59	0.08	72.5	**1.21**	**1.04**–**1.41**	**0.01**	**2.5**	1.16	0.95–1.44	0.15	69.1	1.09	0.96–1.23	0.19	40.7	
Ethnicity																							
Asian	3	1593/1685	**1.14**	**1.04**–**1.25**	**0.01**	0	1.32	0.90–1.94	0.12	81.2	**1.33**	**1.09**–**1.61**	**<0.01**	**0**	**1.32**	**0.99**–**1.76**	**0.06**	**69.7**	**1.14**	**0.99**–**1.33**	**0.08**	**44.8**	
Design																							
Population	3	2613/2691	1.08	0.99–1.18	0.08	0	1.07	0.93–1.24	0.35	0	1.14	0.96–1.36	0.14	0	1.03	0.88–1.19	0.74	34.3	1.11	0.84–1.43	0.40	66.5	
Hospital	2	921/873	**1.16**	**1.01**–**1.33**	**0.03**	0	1.57	0.95–2.57	0.08	77.7	**1.47**	**1.08**–**2.00**	**0.02**	**0**	**1.50**	**1.02**–**2.20**	**0.04**	**66.6**	0.99	0.78–1.26	0.91	19.8	
Location																							
HN	3	2063/2110	1.07	0.97–1.18	0.21	0	1.49	0.83–2.26	0.18	86.7	1.24	0.84–1.83	0.28	63.3	1.18	0.82–1.69	0.38	83.4	0.95	0.80–1.13	0.54	0	
Genotyping																							
AB	3	1955/2009	**1.14**	**1.03**–**1.27**	**0.01**	**0**	1.08	0.90–1.28	0.40	35.3	**1.27**	**1.02**–**1.58**	**0.04**	**0**	1.05	0.85–1.29	0.67	55.3	**1.25**	**1.05**–**1.49**	**0.01**	**0**	
PCR–RFLP	2	1579/1555	1.07	0.97–1.18	0.21	0	1.49	0.83–2.26	0.18	86.7	1.17	0.95–1.44	0.15	63.3	1.40	0.85–2.32	0.18	84.1	0.95	0.80–1.13	0.54	0	
rs2292832 C > T			T vs. C				CT vs. CC				TT vs. CC				CT + TT vs. CC				TT vs. CC + CT				
Total	3	1852/1677	1.04	0.85–1.28	0.71	60.4	1.18	0.67–2.06	0.57	75.8	1.15	0.63–2.12	0.65	77.8	1.18	0.68–2.05	0.55	78.8	0.98	0.81–1.19	0.86	0	
Ethnicity																							
Asian	2	743/547	1.12	0.74–1.69	0.60	76.6	1.37	0.40–4.71	0.62	86.6	1.40	0.42–4.71	0.59	87.1	1.39	0.41–4.69	0.59	88.2	1.05	0.82–1.34	0.73	0	
Genotyping																							
AB	2	743/547	1.12	0.74–1.69	0.60	76.6	1.37	0.40–4.71	0.62	86.6	1.40	0.42–4.71	0.59	87.1	1.39	0.41–4.69	0.59	88.2	1.05	0.82–1.34	0.73	0	
Design																							
Hospital	2	743/547	1.12	0.74–1.69	0.60	76.6	1.37	0.40–4.71	0.62	86.6	1.40	0.42–4.71	0.59	87.1	1.39	0.41–4.69	0.59	88.2	1.05	0.82–1.34	0.73	0	
rs3746444 A > G			G vs. A				AG vs. AA				GG vs. AA				AG + GG vs. AA				GG vs. AA + AG				
Total	2	1579/1555	1.29	0.59–2.80	0.52	95.4	1.22	0.53–2.82	0.64	94.8	1.77	0.43–7.33	0.43	78.6	1.27	0.54–3.01	0.59	95.4	1.68	0.50–5.64	0.40	71.5	

Population: Population controls Hospital: Hospital controls Healthy: Healthy controls.

HN: head and neck.

ABI: Applied Biosystems.

^*^Numbers of comparisons.

^a^Test for heterogeneity.
